# Crystal structure of 2-chloro-1,3-bis­(2,6-diiso­propyl­phen­yl)-1,3,2-di­aza­phospho­lidine 2-oxide

**DOI:** 10.1107/S2056989017005825

**Published:** 2017-04-21

**Authors:** Alex J. Veinot, Arthur D. Hendsbee, Jason D. Masuda

**Affiliations:** a Department of Chemistry, Saint Mary’s University, 923 Robie St., Halifax, Nova Scotia, B3H 3C3, Canada

**Keywords:** crystal structure, *N*-heterocyclic phosphine, NHP, 1,3,2-di­aza­phospho­lidine 2-oxide

## Abstract

The title compound, C_26_H_38_ClN_2_OP, was synthesized by reacting phosphoryl chloride with *N*,*N*′-bis­(2,6-diiso­propyl­phen­yl)ethane-1,2-di­amine in the presence of *N*-methyl­morpholine which acted as an auxilliary base to quench the HCl released as a by-product. The resultant *N*-heterocyclic phosphine five-membered ring adopts a half-chair conformation and features a tetra­coordinate P atom ligated by the chelating di­amine [P—N = 1.6348 (14) and 1.6192 (14) Å], one double-bonded O atom [P1—O1 = 1.4652 (12) Å] and one Cl atom [P1—Cl1 = 2.0592 (7) Å]. The sterically hindered 2,6-diiso­propyl­phenyl (Dipp) groups twist away from the central heterocycle, with torsion angles of −75.66 (19) and 83.39 (19)° for the P—N—C_ar_—C_ar_ links. A number of intra­molecular C—H⋯N, C—H⋯O and C—H⋯Cl close contacts occur. In the crystal, mol­ecules are linked by C—H⋯O hydrogen bonds to generate [010] chains. C—H⋯π inter­actions are also observed.

## Abstract 2

The synthesis, spectroscopic and crystal structure of 2-chloro-1,3-bis­(2,6-diiso­propyl­phen­yl)-1,3,2-di­aza­phospho­lidine 2-oxide are reported.

## Chemical context   

1,3,2-Di­aza­phospho­lidines are a class of *N*-heterocyclic phosphines (NHPs) that feature an N—P—N moiety bridged by a C_2_H_4_ fragment, thus forming a five-membered ring. Derivatives are often substituted by alkyl, aryl, or halogen groups at the phospho­rus position (denoted as position 2), allowing them to serve as both ligands and/or precursors in organometallic chemistry (Gudat, 2010 ▸). The title compound, 2-chloro-1,3-bis­(2,6-diiso­propyl­phen­yl)-1,3,2-di­aza­phospho­li­dine 2-oxide, is closely related to these compounds and its analogs are commonly used as precursor mol­ecules for the synthesis of pharmaceuticals targeted towards immunosuppressants and chemotherapy medications (Gholivand & Mojahed, 2005 ▸). The crystal structure of the title compound is reported herein and features a saturated five-membered NHP substituted at the phospho­rus position by both O and Cl atoms.
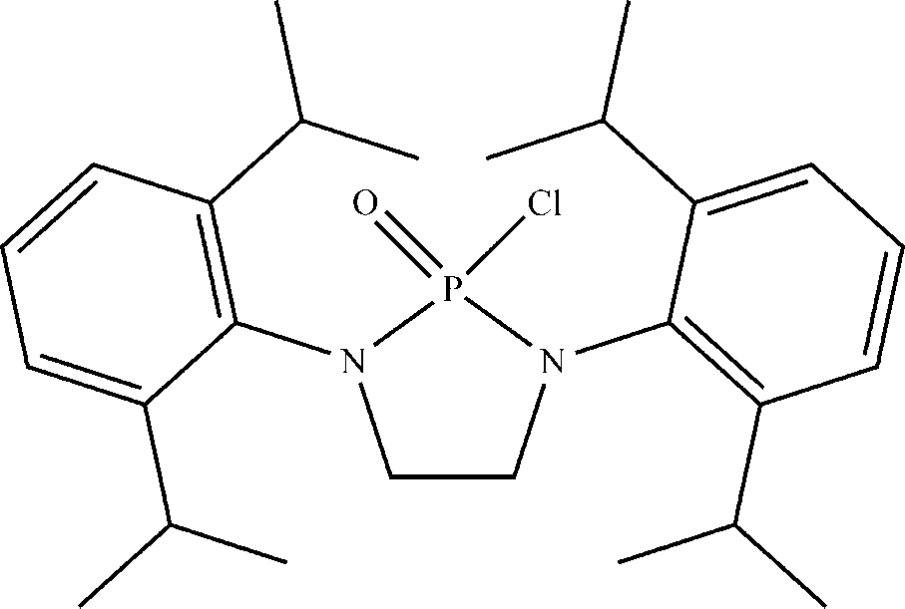


## Structural commentary   

**Figure 1:**
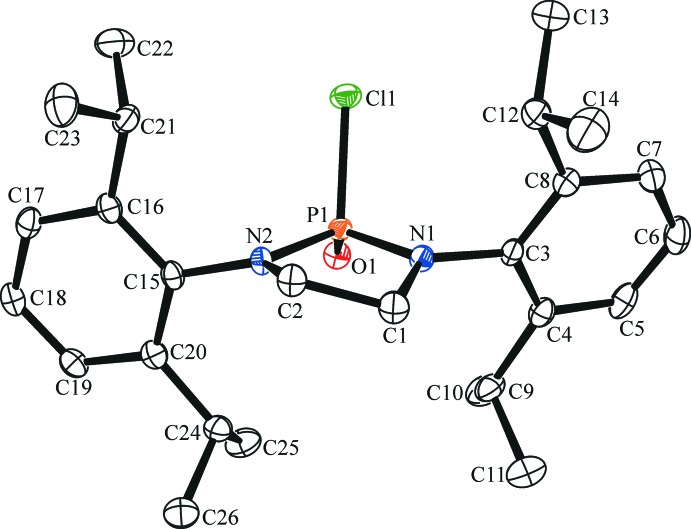
The mol­ecular structure of the title compound, showing 50% probability displacement ellipsoids. H atoms have been omitted for clarity.

The mol­ecular structure of the title compound is shown in Fig. 1 ▸. The title compound crystallizes in the monoclinic space group *P*2_1_/*n* with one mol­ecule present in the asymmetric unit. Bond lengths between the flanking nitro­gen atoms show a statistical difference when compared to each other [P1—N1 = 1.6348 (14) Å and P1—N2 = 1.6192 (14) Å] and is likely caused by the half-chair (or envelope) conformation of the heterocycle at the C2 position. The N—P—N bond angle of 95.60 (7)° deviates significantly from an ideal tetra­hedral geometry. Bond lengths between P1—Cl1 and P1—O1 are 2.0592 (7) and 1.4652 (12) Å, respectively, with a bond angle of 105.51 (5)° for the O—P—Cl atoms. The isopropyl groups are oriented away from the central five-membered ring and lead to intra­molecular short-contact *D*—H⋯*A* inter­actions between methine atoms H9, H12, H21, and H24, and N1 and N2. Intra­molecular short-contact *D*—H⋯*A* inter­actions are also present for Cl1 and O1 atoms and are summarized in Table 1 ▸. The steric demands of the bulky 2,6-diiso­propyl­phenyl groups cause the aromatic rings to twist away from the central five-membered ring with torsion angles of −75.66 (19) and 83.39 (19)° for P1—N1—C3—C4 and P1—N2—C15—C20, respectively. The dihedral angles between the heterocyclic ring (all atoms) and the C3–C8 and C15–C20 aromatic rings are 76.61 (9) and 88.75 (9)°, respectively.

## Supra­molecular features   

**Figure 2:**
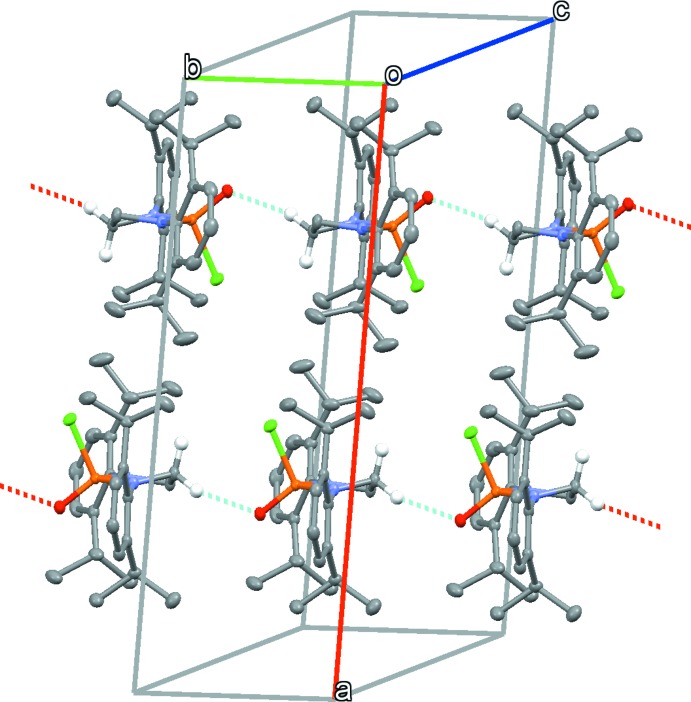
The packing of the title compound, showing the formation of C—H⋯O hydrogen bonds (red and cyan lines).

The crystal of the title compound contains inter­molecular short-contact *D*—H⋯*A* π-inter­actions between C6—H6 and the centroid of the C3–C8 ring of a neighboring mol­ecule (transformation = 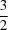 − *x*, −1 + *y*, 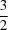 − *z*), with an H⋯centroid distance of 2.740 (3) Å. The isopropyl groups of the flanking aromatic rings also display short contacts for Cl1 and O1, with H⋯Cl distances measuring 2.950 (5) and 3.086 (6) Å between H14*A*⋯Cl1 and H23*B*⋯Cl1, respectively. A significantly short contact of 2.357 (2) Å occurs for H2*A*⋯O1. A distance this small is likely indicative of C—H⋯O hydrogen bonding (Fig. 2 ▸, Table 1 ▸) accepted by the O atom from a neighbouring ethyl­ene bridge related by symmetry (transformation = *x*, *y* − 1, *z*).

## Database survey   

A search of the Cambridge Structural Database (Groom *et al.*, 2016 ▸) produced two matches for 1,3,2-(di­aryl­amino)­phospho­lidine-2-oxide-2-halide derivatives; 1,3-di(*p*-tol­yl)-2-chloro-1,3,2-di­aza­phospho­lidine-2-oxide (*p*-tolyl = 4-methyl­phen­yl), and the analogous fluorine derivative (CSD identifiers WASFEC and SIVJEN, respectively; Gholivand & Mojahed, 2005 ▸). One other closely related bicyclic structure was found (CSD identifier NUMBAY; Koeller *et al.*, 1995 ▸), which features *N*-benzyl substituents and a cyclo­hexyl ring fused to the bridging ethyl­ene C atoms.

## Synthesis and crystallization   

The synthesis of the title compound was achieved using a similar method as used for 2-chloro-1,3-bis­(2,6-diiso­propyl­phen­yl)-1,3,2-di­aza­phospho­lidine (Caputo *et al.*, 2008 ▸), except phosphoryl chloride was used instead of phospho­rus trichloride. In a 200 ml Schlenk flask, 1.142 g (3.00 mmol, 1 eq.) of *N*,*N*′-bis­(2,6-diiso­propyl­phen­yl)ethane-1,2-di­amine were dissolved in 45 ml of THF producing a colourless solution. Separately 0.478 g (3.11 mmol, 1.04 eq.) of phosphoryl chloride and 0.959 g (9.48 mmol, 3.16 eq.) of *N*-methyl­morpholine were dissolved in 75 ml of THF producing a colourless solution, and transferred to a 125 ml pressure-equalizing dropping funnel. The di­amine solution was cooled to 195 K using a liquid nitro­gen/acetone bath and monitored using a thermocouple, and once cold (*ca* 10 minutes) the phosphoryl chloride mixture was added dropwise to the di­amine solution over 30 minutes. Once the addition was complete, the colourless reaction mixture was left to stir at 195 K for 60 minutes, after which it was allowed to warm to room temperature and left to stir for two days at room temperature. The reaction was monitored by ^31^P{^1^H} NMR spectroscopy, and became pale yellow in colour with a slight amount of colourless precipitate as it proceeded. Once the starting material was completely consumed, the reaction mixture was dried *in vacuo* to give a pale-yellow coloured solid. Extraction of this solid with 50 ml of a 3:2 mixture of penta­ne:THF produced the desired product as a pale-yellow coloured solution following filtration through Celite, which when dried *in vacuo* afforded 0.919 g (66%) of the desired product as a faintly yellow coloured powder. Crystals of the product in the form of colourless blocks were obtained by concentrating the filtrate and storing in a 238 K freezer overnight.

^1^H NMR (CDCl_3_): δ 7.32 (*t*, ^3^ *J*_HH_ = 7.6 Hz, 2H, *p*-Dipp), 7.21 (*m*, ^3^ *J*_HH_ = 7.4 Hz, 4H, *m*-Dipp), 3.86–3.50 (*m*, 8H, *i*Pr—CH, NHC—CH_2_), 1.38 (*d*, ^3^ *J*_HH_ = 6.8 Hz, 6H, *i*Pr—CH_3_), 1.35 (*d*, ^3^ *J*_HH_ = 6.8 Hz, 6H, *i*Pr—CH_3_), 1.28 (*d*, ^3^ *J*_HH_ = 6.9 Hz, 6H, *i*Pr—CH_3_), 1.26 ppm (*d*, ^3^ *J*_HH_ = 6.9 Hz, 6H, iPr—CH_3_). ^13^C{^1^H} NMR (CDCl_3_): δ 149.8, 149.6, 131.8, 129.1, 125.0, 124.9, 48.8, 29.0, 25.9, 24.5, 23.9 ppm. ^31^P{^1^H} NMR (CDCl_3_): δ 15.1 ppm. IR (KBr pellet): ν 3068 (*w*), 2967 (*s*), 2929 (*m*), 2869 (*m*), 1681 (*w*), 1588 (*w*), 1464 (*s*), 1448 (*s*), 1383 (*w*), 1368 (*w*), 1348 (*w*) 1323 (*m*), 1268 (*s*), 1217 (*w*), 1194 (*w*), 1106 (*m*), 1093 (*m*), 1077 (*m*), 1056 (*m*), 1043 (*w*), 934 (*w*), 860 (*w*), 803 (*s*), 756 (*w*), 747 (*w*), 733 (*w*), 648 (*w*), 592 (*w*), 575 (*w*), 558 (*w*), 544 (*w*), 496 (*s*) 466 (*w*), 437 (*w*), 412 cm^−1^ (*w*). m.p. 509.7–511.0 K.

## Refinement   

Crystal data, data collection and structure refinement details are summarized in Table 2 ▸. H atoms were included in geometrically idealized positions and refined using a riding model [C—H = 0.95–0.99; *U*_iso_(H) = 1.2–1.5*U*_eq_(C)]. The methyl H atoms were allowed to rotate, but not to tip, to best fit the electron density.

## Supplementary Material

Crystal structure: contains datablock(s) I. DOI: 10.1107/S2056989017005825/hb7671sup1.cif

Structure factors: contains datablock(s) I. DOI: 10.1107/S2056989017005825/hb7671Isup2.hkl

Supporting information file. DOI: 10.1107/S2056989017005825/hb7671Isup3.cml

CCDC reference: 1544709

Additional supporting information:  crystallographic information; 3D view; checkCIF report

## Figures and Tables

**Table 1:** Hydrogen-bond geometry (Å, °)

*D*—H⋯*A*	*D*—H	H⋯*A*	*D*⋯*A*	*D*—H⋯*A*
C12—H12⋯Cl1	1.00	2.91	3.543 (2)	122
C21—H21⋯Cl1	1.00	2.88	3.6006 (19)	130
C9—H9⋯O1	1.00	2.63	3.273 (2)	122
C25—H25*C*⋯O1	0.98	2.61	3.407 (2)	138
C9—H9⋯N1	1.00	2.43	2.927 (2)	110
C12—H12⋯N1	1.00	2.41	2.904 (2)	110
C21—H21⋯N2	1.00	2.42	2.930 (2)	111
C24—H24⋯N2	1.00	2.41	2.915 (2)	110
C2—H2*A*⋯O1^i^	0.99	2.36	3.319 (2)	164

Symmetry code: (i) 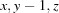.

**Table 2:** Experimental details

Crystal data	
Chemical formula	C_26_H_38_ClN_2_OP
*M*_r_	461.00
Crystal system, space group	Monoclinic, *P*2_1_/*n*
Temperature (K)	104
*a*, *b*, *c* (Å)	19.984 (3), 6.6328 (11), 20.140 (3)
β (°)	106.818 (2)
*V* (Å^3^)	2555.4 (7)
*Z*	4
Radiation type	Mo *K*α
μ (mm^−1^)	0.23
Crystal size (mm)	0.25 × 0.21 × 0.17

Data collection	
Diffractometer	Siemens/Bruker APEXII
Absorption correction	Multi-scan (*SADABS*; Bruker, 2008 ▸)
*T*_min_, *T*_max_	0.578, 0.746
No. of measured, independent and observed [*I* > 2σ(*I*)] reflections	30194, 6307, 4640
*R*_int_	0.073
(sin θ/λ)_max_ (Å^−1^)	0.669

Refinement	
*R*[*F* ^2^ > 2σ(*F* ^2^)], *wR*(*F* ^2^), *S*	0.043, 0.109, 1.04
No. of reflections	6307
No. of parameters	288
H-atom treatment	H-atom parameters constrained
Δρ_max_, Δρ_min_ (e Å^−3^)	0.43, −0.33

Computer programs: *APEX2* and *SAINT* (Bruker, 2008 ▸), *SHELXT2014* (Sheldrick, 2015*a* ▸), *SHELXL2016* (Sheldrick, 2015*b* ▸), *ORTEP-3 for Windows* (Farrugia, 2012 ▸), *PLATON* (Spek, 2009 ▸) and *publCIF* (Westrip, 2010 ▸).

## supplementary crystallographic information

### Crystal data

**Table:**

C_26_H_38_ClN_2_OP	*F*(000) = 992
*M**_r_* = 461.00	*D*_x_ = 1.198 Mg m^−^^3^
Monoclinic, *P*2_1_/*n*	Mo *K*α radiation, λ = 0.71073 Å
*a* = 19.984 (3) Å	Cell parameters from 4093 reflections
*b* = 6.6328 (11) Å	θ = 2.5–24.5°
*c* = 20.140 (3) Å	µ = 0.23 mm^−^^1^
β = 106.818 (2)°	*T* = 104 K
*V* = 2555.4 (7) Å^3^	Block, colourless
*Z* = 4	0.25 × 0.21 × 0.17 mm

### Data collection

**Table:**

Siemens/Bruker APEXII diffractometer	4640 reflections with *I* > 2σ(*I*)
Detector resolution: 66 pixels mm^-1^	*R*_int_ = 0.073
φ and ω scans	θ_max_ = 28.4°, θ_min_ = 2.1°
Absorption correction: multi-scan (SADABS; Bruker, 2008)	*h* = −26→26
*T*_min_ = 0.578, *T*_max_ = 0.746	*k* = −8→8
30194 measured reflections	*l* = −26→26
6307 independent reflections	

### Refinement

**Table:**

Refinement on *F*^2^	Primary atom site location: structure-invariant direct methods
Least-squares matrix: full	Hydrogen site location: inferred from neighbouring sites
*R*[*F*^2^ > 2σ(*F*^2^)] = 0.043	H-atom parameters constrained
*wR*(*F*^2^) = 0.109	*w* = 1/[σ^2^(*F*_o_^2^) + (0.0435*P*)^2^ + 0.5837*P*] where *P* = (*F*_o_^2^ + 2*F*_c_^2^)/3
*S* = 1.04	(Δ/σ)_max_ = 0.001
6307 reflections	Δρ_max_ = 0.43 e Å^−^^3^
288 parameters	Δρ_min_ = −0.33 e Å^−^^3^
0 restraints	

### Special details

**Table:**

Geometry. All esds (except the esd in the dihedral angle between two l.s. planes) are estimated using the full covariance matrix. The cell esds are taken into account individually in the estimation of esds in distances, angles and torsion angles; correlations between esds in cell parameters are only used when they are defined by crystal symmetry. An approximate (isotropic) treatment of cell esds is used for estimating esds involving l.s. planes.

### Fractional atomic coordinates and isotropic or equivalent isotropic displacement parameters (Å^2^)

**Table:**

	*x*	*y*	*z*	*U*_iso_*/*U*_eq_	
CL1	0.60905 (2)	0.77730 (7)	0.42592 (2)	0.02113 (12)	
P1	0.70785 (2)	0.65590 (7)	0.46135 (2)	0.01383 (11)	
O1	0.75532 (6)	0.81335 (17)	0.45109 (6)	0.0181 (3)	
N1	0.71214 (7)	0.5695 (2)	0.53850 (7)	0.0149 (3)	
N2	0.70937 (7)	0.4369 (2)	0.42630 (7)	0.0147 (3)	
C1	0.72101 (10)	0.3487 (3)	0.54424 (9)	0.0196 (4)	
H1A	0.692450	0.290717	0.572321	0.024*	
H1B	0.770625	0.312993	0.566036	0.024*	
C2	0.69615 (9)	0.2713 (3)	0.46958 (9)	0.0183 (4)	
H2A	0.722531	0.149108	0.464119	0.022*	
H2B	0.645730	0.237947	0.456523	0.022*	
C3	0.71736 (9)	0.6963 (2)	0.59765 (8)	0.0149 (3)	
C4	0.78155 (9)	0.7887 (3)	0.63099 (9)	0.0189 (4)	
C5	0.78352 (10)	0.9197 (3)	0.68551 (9)	0.0245 (4)	
H5	0.825951	0.987103	0.708089	0.029*	
C6	0.72518 (11)	0.9539 (3)	0.70750 (10)	0.0273 (4)	
H6	0.727763	1.044273	0.744756	0.033*	
C7	0.66305 (10)	0.8570 (3)	0.67543 (9)	0.0256 (4)	
H7	0.623418	0.879569	0.691479	0.031*	
C8	0.65767 (9)	0.7266 (3)	0.61984 (9)	0.0192 (4)	
C9	0.84815 (9)	0.7422 (3)	0.61222 (9)	0.0223 (4)	
H9	0.834851	0.662355	0.568225	0.027*	
C10	0.88538 (10)	0.9329 (3)	0.59894 (11)	0.0314 (5)	
H10A	0.926952	0.895280	0.585271	0.047*	
H10B	0.899393	1.014211	0.641348	0.047*	
H10C	0.853666	1.011382	0.561687	0.047*	
C11	0.89651 (10)	0.6108 (3)	0.66826 (11)	0.0309 (5)	
H11A	0.938829	0.578975	0.655073	0.046*	
H11B	0.872366	0.485491	0.673151	0.046*	
H11C	0.909329	0.683572	0.712499	0.046*	
C12	0.58966 (9)	0.6166 (3)	0.58712 (10)	0.0247 (4)	
H12	0.593698	0.549291	0.544010	0.030*	
C13	0.52710 (11)	0.7590 (4)	0.56644 (11)	0.0407 (6)	
H13A	0.535887	0.864893	0.536069	0.061*	
H13B	0.520044	0.820457	0.608159	0.061*	
H13C	0.485179	0.683169	0.541819	0.061*	
C14	0.57816 (11)	0.4518 (4)	0.63611 (11)	0.0373 (5)	
H14A	0.535680	0.375892	0.613176	0.056*	
H14B	0.573101	0.513955	0.678539	0.056*	
H14C	0.618349	0.360144	0.647930	0.056*	
C15	0.72566 (9)	0.4045 (2)	0.36189 (9)	0.0155 (4)	
C16	0.67152 (9)	0.3889 (2)	0.29983 (9)	0.0172 (4)	
C17	0.68940 (10)	0.3713 (3)	0.23813 (9)	0.0197 (4)	
H17	0.653475	0.361779	0.195399	0.024*	
C18	0.75855 (10)	0.3673 (3)	0.23807 (9)	0.0203 (4)	
H18	0.769988	0.359264	0.195532	0.024*	
C19	0.81087 (10)	0.3752 (3)	0.30022 (9)	0.0196 (4)	
H19	0.858228	0.368268	0.299803	0.023*	
C20	0.79627 (9)	0.3931 (3)	0.36346 (9)	0.0173 (4)	
C21	0.59508 (9)	0.3881 (3)	0.29800 (9)	0.0188 (4)	
H21	0.592775	0.410635	0.346382	0.023*	
C22	0.55404 (10)	0.5564 (3)	0.25268 (10)	0.0295 (5)	
H22A	0.555477	0.537843	0.204835	0.044*	
H22B	0.574841	0.686840	0.270126	0.044*	
H22C	0.505386	0.552822	0.253905	0.044*	
C23	0.56189 (11)	0.1827 (3)	0.27379 (11)	0.0306 (5)	
H23A	0.513339	0.182330	0.275382	0.046*	
H23B	0.588161	0.076821	0.304337	0.046*	
H23C	0.562927	0.157514	0.226146	0.046*	
C24	0.85570 (9)	0.3942 (3)	0.43053 (9)	0.0195 (4)	
H24	0.834454	0.399067	0.469768	0.023*	
C25	0.90220 (10)	0.5797 (3)	0.43636 (10)	0.0270 (4)	
H25A	0.925377	0.576285	0.399635	0.041*	
H25B	0.937539	0.580306	0.481732	0.041*	
H25C	0.873536	0.701785	0.431455	0.041*	
C26	0.89855 (11)	0.2004 (3)	0.43818 (10)	0.0289 (5)	
H26A	0.867806	0.083879	0.435911	0.043*	
H26B	0.934841	0.201006	0.482942	0.043*	
H26C	0.920487	0.191919	0.400596	0.043*	

### Atomic displacement parameters (Å^2^)

**Table:**

	*U*^11^	*U*^22^	*U*^33^	*U*^12^	*U*^13^	*U*^23^
CL1	0.0179 (2)	0.0199 (2)	0.0225 (2)	0.00405 (17)	0.00101 (17)	0.00066 (17)
P1	0.0146 (2)	0.0125 (2)	0.0134 (2)	0.00099 (17)	0.00254 (17)	0.00000 (17)
O1	0.0209 (7)	0.0144 (6)	0.0190 (6)	−0.0012 (5)	0.0058 (5)	0.0009 (5)
N1	0.0180 (8)	0.0122 (7)	0.0139 (7)	0.0010 (6)	0.0035 (6)	−0.0002 (6)
N2	0.0183 (8)	0.0135 (7)	0.0129 (7)	−0.0003 (6)	0.0054 (6)	−0.0011 (6)
C1	0.0260 (10)	0.0132 (9)	0.0198 (9)	0.0013 (7)	0.0068 (8)	0.0021 (7)
C2	0.0230 (9)	0.0135 (9)	0.0187 (9)	−0.0013 (7)	0.0066 (7)	0.0008 (7)
C3	0.0191 (9)	0.0131 (8)	0.0111 (8)	0.0009 (7)	0.0024 (7)	−0.0003 (6)
C4	0.0231 (9)	0.0170 (9)	0.0153 (8)	−0.0007 (7)	0.0034 (7)	0.0017 (7)
C5	0.0304 (11)	0.0233 (10)	0.0156 (9)	−0.0080 (8)	−0.0001 (8)	−0.0017 (8)
C6	0.0406 (12)	0.0220 (10)	0.0185 (9)	0.0009 (9)	0.0074 (9)	−0.0056 (8)
C7	0.0286 (11)	0.0297 (11)	0.0196 (10)	0.0059 (9)	0.0088 (8)	−0.0023 (8)
C8	0.0197 (9)	0.0214 (9)	0.0157 (9)	0.0017 (7)	0.0039 (7)	0.0006 (7)
C9	0.0166 (9)	0.0291 (11)	0.0185 (9)	−0.0024 (8)	0.0008 (7)	0.0016 (8)
C10	0.0215 (10)	0.0386 (12)	0.0308 (11)	−0.0073 (9)	0.0023 (9)	0.0077 (9)
C11	0.0242 (11)	0.0321 (12)	0.0318 (11)	−0.0004 (9)	0.0007 (9)	0.0051 (9)
C12	0.0181 (10)	0.0374 (12)	0.0192 (9)	−0.0009 (8)	0.0062 (8)	−0.0030 (8)
C13	0.0225 (11)	0.0671 (17)	0.0309 (12)	0.0118 (11)	0.0052 (9)	−0.0062 (11)
C14	0.0280 (12)	0.0538 (15)	0.0309 (12)	−0.0152 (10)	0.0096 (9)	0.0020 (10)
C15	0.0209 (9)	0.0115 (8)	0.0155 (8)	0.0002 (7)	0.0072 (7)	−0.0014 (7)
C16	0.0217 (9)	0.0119 (8)	0.0191 (9)	−0.0007 (7)	0.0076 (7)	−0.0019 (7)
C17	0.0253 (10)	0.0162 (9)	0.0160 (9)	−0.0010 (7)	0.0035 (8)	−0.0017 (7)
C18	0.0275 (10)	0.0175 (9)	0.0190 (9)	−0.0004 (8)	0.0116 (8)	−0.0024 (7)
C19	0.0211 (9)	0.0159 (9)	0.0237 (9)	0.0004 (7)	0.0097 (8)	−0.0007 (7)
C20	0.0203 (9)	0.0128 (8)	0.0196 (9)	−0.0011 (7)	0.0070 (7)	−0.0004 (7)
C21	0.0177 (9)	0.0220 (10)	0.0151 (9)	−0.0019 (7)	0.0021 (7)	−0.0018 (7)
C22	0.0210 (10)	0.0392 (12)	0.0280 (11)	0.0049 (9)	0.0068 (9)	0.0092 (9)
C23	0.0312 (11)	0.0328 (12)	0.0304 (11)	−0.0131 (9)	0.0130 (9)	−0.0099 (9)
C24	0.0163 (9)	0.0225 (10)	0.0199 (9)	0.0013 (7)	0.0056 (7)	0.0004 (7)
C25	0.0213 (10)	0.0251 (10)	0.0305 (11)	−0.0029 (8)	0.0010 (8)	0.0019 (8)
C26	0.0305 (11)	0.0273 (11)	0.0270 (11)	0.0081 (9)	0.0052 (9)	0.0032 (9)

### Geometric parameters (Å, º)

**Table:**

CL1—P1	2.0592 (7)	C13—H13A	0.9800
P1—O1	1.4652 (12)	C13—H13B	0.9800
P1—N2	1.6192 (14)	C13—H13C	0.9800
P1—N1	1.6348 (14)	C14—H14A	0.9800
N1—C3	1.437 (2)	C14—H14B	0.9800
N1—C1	1.476 (2)	C14—H14C	0.9800
N2—C15	1.442 (2)	C15—C16	1.400 (2)
N2—C2	1.473 (2)	C15—C20	1.404 (2)
C1—C2	1.529 (2)	C16—C17	1.394 (2)
C1—H1A	0.9900	C16—C21	1.517 (2)
C1—H1B	0.9900	C17—C18	1.382 (3)
C2—H2A	0.9900	C17—H17	0.9500
C2—H2B	0.9900	C18—C19	1.380 (3)
C3—C8	1.404 (2)	C18—H18	0.9500
C3—C4	1.405 (2)	C19—C20	1.392 (2)
C4—C5	1.392 (2)	C19—H19	0.9500
C4—C9	1.517 (2)	C20—C24	1.519 (2)
C5—C6	1.380 (3)	C21—C22	1.523 (3)
C5—H5	0.9500	C21—C23	1.532 (3)
C6—C7	1.381 (3)	C21—H21	1.0000
C6—H6	0.9500	C22—H22A	0.9800
C7—C8	1.394 (2)	C22—H22B	0.9800
C7—H7	0.9500	C22—H22C	0.9800
C8—C12	1.515 (3)	C23—H23A	0.9800
C9—C11	1.529 (3)	C23—H23B	0.9800
C9—C10	1.530 (3)	C23—H23C	0.9800
C9—H9	1.0000	C24—C25	1.526 (3)
C10—H10A	0.9800	C24—C26	1.527 (3)
C10—H10B	0.9800	C24—H24	1.0000
C10—H10C	0.9800	C25—H25A	0.9800
C11—H11A	0.9800	C25—H25B	0.9800
C11—H11B	0.9800	C25—H25C	0.9800
C11—H11C	0.9800	C26—H26A	0.9800
C12—C13	1.526 (3)	C26—H26B	0.9800
C12—C14	1.534 (3)	C26—H26C	0.9800
C12—H12	1.0000		
			
O1—P1—N2	118.91 (7)	C12—C13—H13A	109.5
O1—P1—N1	121.71 (7)	C12—C13—H13B	109.5
N2—P1—N1	95.60 (7)	H13A—C13—H13B	109.5
O1—P1—CL1	105.51 (5)	C12—C13—H13C	109.5
N2—P1—CL1	109.68 (6)	H13A—C13—H13C	109.5
N1—P1—CL1	104.35 (5)	H13B—C13—H13C	109.5
C3—N1—C1	122.51 (14)	C12—C14—H14A	109.5
C3—N1—P1	123.66 (12)	C12—C14—H14B	109.5
C1—N1—P1	113.22 (11)	H14A—C14—H14B	109.5
C15—N2—C2	123.16 (13)	C12—C14—H14C	109.5
C15—N2—P1	124.30 (11)	H14A—C14—H14C	109.5
C2—N2—P1	112.45 (11)	H14B—C14—H14C	109.5
N1—C1—C2	105.00 (13)	C16—C15—C20	121.87 (15)
N1—C1—H1A	110.7	C16—C15—N2	119.78 (15)
C2—C1—H1A	110.7	C20—C15—N2	118.34 (15)
N1—C1—H1B	110.7	C17—C16—C15	118.09 (16)
C2—C1—H1B	110.7	C17—C16—C21	119.61 (16)
H1A—C1—H1B	108.8	C15—C16—C21	122.30 (15)
N2—C2—C1	105.58 (13)	C18—C17—C16	121.10 (17)
N2—C2—H2A	110.6	C18—C17—H17	119.5
C1—C2—H2A	110.6	C16—C17—H17	119.5
N2—C2—H2B	110.6	C19—C18—C17	119.58 (16)
C1—C2—H2B	110.6	C19—C18—H18	120.2
H2A—C2—H2B	108.8	C17—C18—H18	120.2
C8—C3—C4	121.87 (15)	C18—C19—C20	121.92 (17)
C8—C3—N1	118.87 (15)	C18—C19—H19	119.0
C4—C3—N1	119.25 (15)	C20—C19—H19	119.0
C5—C4—C3	117.57 (16)	C19—C20—C15	117.35 (16)
C5—C4—C9	119.80 (16)	C19—C20—C24	119.82 (16)
C3—C4—C9	122.55 (16)	C15—C20—C24	122.81 (15)
C6—C5—C4	121.46 (18)	C16—C21—C22	112.06 (15)
C6—C5—H5	119.3	C16—C21—C23	110.57 (15)
C4—C5—H5	119.3	C22—C21—C23	110.65 (16)
C5—C6—C7	120.12 (17)	C16—C21—H21	107.8
C5—C6—H6	119.9	C22—C21—H21	107.8
C7—C6—H6	119.9	C23—C21—H21	107.8
C6—C7—C8	120.96 (18)	C21—C22—H22A	109.5
C6—C7—H7	119.5	C21—C22—H22B	109.5
C8—C7—H7	119.5	H22A—C22—H22B	109.5
C7—C8—C3	117.96 (16)	C21—C22—H22C	109.5
C7—C8—C12	119.95 (16)	H22A—C22—H22C	109.5
C3—C8—C12	122.05 (16)	H22B—C22—H22C	109.5
C4—C9—C11	110.16 (15)	C21—C23—H23A	109.5
C4—C9—C10	112.46 (16)	C21—C23—H23B	109.5
C11—C9—C10	111.41 (16)	H23A—C23—H23B	109.5
C4—C9—H9	107.5	C21—C23—H23C	109.5
C11—C9—H9	107.5	H23A—C23—H23C	109.5
C10—C9—H9	107.5	H23B—C23—H23C	109.5
C9—C10—H10A	109.5	C20—C24—C25	111.98 (15)
C9—C10—H10B	109.5	C20—C24—C26	110.92 (15)
H10A—C10—H10B	109.5	C25—C24—C26	111.09 (15)
C9—C10—H10C	109.5	C20—C24—H24	107.5
H10A—C10—H10C	109.5	C25—C24—H24	107.5
H10B—C10—H10C	109.5	C26—C24—H24	107.5
C9—C11—H11A	109.5	C24—C25—H25A	109.5
C9—C11—H11B	109.5	C24—C25—H25B	109.5
H11A—C11—H11B	109.5	H25A—C25—H25B	109.5
C9—C11—H11C	109.5	C24—C25—H25C	109.5
H11A—C11—H11C	109.5	H25A—C25—H25C	109.5
H11B—C11—H11C	109.5	H25B—C25—H25C	109.5
C8—C12—C13	112.45 (17)	C24—C26—H26A	109.5
C8—C12—C14	110.23 (15)	C24—C26—H26B	109.5
C13—C12—C14	110.69 (17)	H26A—C26—H26B	109.5
C8—C12—H12	107.8	C24—C26—H26C	109.5
C13—C12—H12	107.8	H26A—C26—H26C	109.5
C14—C12—H12	107.8	H26B—C26—H26C	109.5
			
O1—P1—N1—C3	46.64 (16)	C5—C4—C9—C11	71.0 (2)
N2—P1—N1—C3	175.81 (13)	C3—C4—C9—C11	−105.6 (2)
CL1—P1—N1—C3	−72.20 (13)	C5—C4—C9—C10	−53.9 (2)
O1—P1—N1—C1	−124.54 (12)	C3—C4—C9—C10	129.42 (18)
N2—P1—N1—C1	4.63 (13)	C7—C8—C12—C13	52.4 (2)
CL1—P1—N1—C1	116.61 (11)	C3—C8—C12—C13	−130.01 (18)
O1—P1—N2—C15	−31.45 (16)	C7—C8—C12—C14	−71.7 (2)
N1—P1—N2—C15	−162.56 (14)	C3—C8—C12—C14	106.0 (2)
CL1—P1—N2—C15	90.01 (14)	C2—N2—C15—C16	88.2 (2)
O1—P1—N2—C2	145.24 (11)	P1—N2—C15—C16	−95.46 (18)
N1—P1—N2—C2	14.13 (13)	C2—N2—C15—C20	−93.0 (2)
CL1—P1—N2—C2	−93.30 (11)	P1—N2—C15—C20	83.39 (19)
C3—N1—C1—C2	168.22 (14)	C20—C15—C16—C17	−3.1 (3)
P1—N1—C1—C2	−20.48 (17)	N2—C15—C16—C17	175.75 (15)
C15—N2—C2—C1	149.38 (15)	C20—C15—C16—C21	176.14 (16)
P1—N2—C2—C1	−27.36 (17)	N2—C15—C16—C21	−5.0 (2)
N1—C1—C2—N2	28.46 (18)	C15—C16—C17—C18	0.6 (3)
C1—N1—C3—C8	−86.2 (2)	C21—C16—C17—C18	−178.65 (16)
P1—N1—C3—C8	103.40 (17)	C16—C17—C18—C19	1.9 (3)
C1—N1—C3—C4	94.7 (2)	C17—C18—C19—C20	−1.9 (3)
P1—N1—C3—C4	−75.66 (19)	C18—C19—C20—C15	−0.5 (3)
C8—C3—C4—C5	−2.7 (3)	C18—C19—C20—C24	177.98 (16)
N1—C3—C4—C5	176.29 (15)	C16—C15—C20—C19	3.0 (3)
C8—C3—C4—C9	173.98 (16)	N2—C15—C20—C19	−175.83 (15)
N1—C3—C4—C9	−7.0 (2)	C16—C15—C20—C24	−175.40 (16)
C3—C4—C5—C6	1.8 (3)	N2—C15—C20—C24	5.8 (2)
C9—C4—C5—C6	−175.06 (17)	C17—C16—C21—C22	−58.1 (2)
C4—C5—C6—C7	0.1 (3)	C15—C16—C21—C22	122.71 (18)
C5—C6—C7—C8	−1.2 (3)	C17—C16—C21—C23	65.9 (2)
C6—C7—C8—C3	0.2 (3)	C15—C16—C21—C23	−113.34 (19)
C6—C7—C8—C12	177.92 (17)	C19—C20—C24—C25	65.7 (2)
C4—C3—C8—C7	1.8 (3)	C15—C20—C24—C25	−115.96 (19)
N1—C3—C8—C7	−177.25 (16)	C19—C20—C24—C26	−59.0 (2)
C4—C3—C8—C12	−175.87 (16)	C15—C20—C24—C26	119.31 (18)
N1—C3—C8—C12	5.1 (2)		

### Hydrogen-bond geometry (Å, º)

**Table:**

*D*—H···*A*	*D*—H	H···*A*	*D*···*A*	*D*—H···*A*
C12—H12···Cl1	1.00	2.91	3.543 (2)	122
C21—H21···Cl1	1.00	2.88	3.6006 (19)	130
C9—H9···O1	1.00	2.63	3.273 (2)	122
C25—H25*C*···O1	0.98	2.61	3.407 (2)	138
C9—H9···N1	1.00	2.43	2.927 (2)	110
C12—H12···N1	1.00	2.41	2.904 (2)	110
C21—H21···N2	1.00	2.42	2.930 (2)	111
C24—H24···N2	1.00	2.41	2.915 (2)	110
C2—H2*A*···O1^i^	0.99	2.36	3.319 (2)	164

Symmetry code: (i) *x*, *y*−1, *z*.
